# Deep venous thrombosis and acute pericarditis associated with severe acute respiratory syndrome coronavirus 2 infection in a Congolese infant with sickle cell disease: a case report

**DOI:** 10.1186/s13256-022-03459-8

**Published:** 2022-08-10

**Authors:** Toni Kasole Lubala, Tony Kayembe-Kitenge, Paul Makinko, Luguette Kalenga, Hénoch Kachil, Axel Kayembe, Augustin Mutombo, Mick Shongo

**Affiliations:** 1 Department of Pediatrics, University of Lubumbashi, Lubumbashi, Democratic Republic of the Congo; 2 Department of Pediatrics, CMDC Clinic, Lubumbashi, Democratic Republic of the Congo; 3 Unit of Toxicology, University of Lubumbashi, Lubumbashi, Democratic Republic of the Congo; 4 High Institute of Medical Techniques (ISTM), Lubumbashi, Democratic Republic of the Congo; 5 Center for Environment and Health, Department of Public Health and Primary Care, KU Leuven, Leuven, Belgium; 6 Department of Radiology, CMDC Clinic, Lubumbashi, Democratic Republic of the Congo

**Keywords:** Thrombosis, Pericarditis, SARS-CoV-2, Sickle cell disease, Congo

## Abstract

**Background:**

Since the beginning of the pandemic, no severe pediatric coronavirus disease 2019 cases have been described in Congo.

**Case:**

We studied a 3-month-old male child of Congolese origin who was admitted to the pediatric department with 7-day history of fever, unilateral lower leg swelling, and dyspnea. There was no known history of contact with a coronavirus disease 2019 patient, and all the family members were asymptomatic. Nasopharyngeal swabs done at admission did not detect severe acute respiratory syndrome coronavirus 2. However, serology tests for severe acute respiratory syndrome coronavirus 2 antibodies were positive for immunoglobulin M and negative for immunoglobulin G. Hemoglobin electrophoresis showed hemoglobin A1, hemoglobin A2, hemoglobin F, and hemoglobin S of 46.2%, 2.5%, 19.9%, and 38.4%, respectively. Chest X-ray showed retrocardiac pneumonia in the left lung, and Doppler ultrasound of the left lower limb showed a recent total femoropopliteal venous thrombosis. At day 10 of hospitalization, our patient had classical signs of cardiac tamponade with a voluminous pericardial effusion seen on echocardiographic examination and elevated C-reactive protein, compatible with a diagnosis of constrictive pericarditis. To the best of the authors’ knowledge, this is the first report of a case of plausible severe acute respiratory syndrome coronavirus 2 infection associated with venous thrombosis and acute pericarditis in Congo.

**Conclusion:**

We hypothesized that this case of venous thrombosis and acute pericarditis in a Congolese child with heterozygous sickle cell disease was related to severe acute respiratory syndrome coronavirus 2 infection.

## Introduction

The severe acute respiratory syndrome coronavirus 2 (SARS-CoV-2) pandemic is a public health emergency of international concern. During the first wave of the epidemic, published data suggested that coronavirus disease 2019 (COVID-19) primarily affects adults while infants were variably but generally mildly affected [1]. Moreover, it has been claimed that the high-risk populations for COVID-19 include elderly individuals and people with severe comorbidities such as diabetes mellitus and high blood pressure [2]. Nevertheless, some case series from China and Europe have reported critical illness in children who present for medical attention with severe or critical illness, requiring hospitalization and intensive care support [1]. Since the beginning of the pandemic in the Democratic Republic of the Congo (DRC) at the end of March 2020, no severe COVID-19 pediatric infections have been described in its second largest city, Lubumbashi, DRC.

However, during the second wave of the epidemic, because of new SARS-CoV-2 variants and after the reopening of primary and secondary schools, more pediatric patients began to present for pediatric consultations for symptomatic COVID-19. Since then, limited data have been published on SARS-CoV-2 infection in young children in the DRC. Here, we report a pediatric case of COVID-19-related thrombotic complication observed at the end of March 2021 in a Congolese patient. We present herein the severity and atypical manifestations of COVID-19 among young children with sickle cell disease (SCD).

## Case presentation

A previously healthy 3-month-old male child of Congolese origin was admitted to the pediatric department with a seven-day history of fever, unilateral lower leg swelling and dyspnea. There was no known history of contact with a COVID-19 patient and all the family members were asymptomatic.

On physical examination, he was afebrile but dehydrated, prostrated, and with a painful tumefaction of the left lower limb. We noted tachypnea and hypoxemia, with saturation of 93.5%. The infant’s body temperature was 36.5 °C, and his heart rate was 140 beats per minute, with a respiratory rate of 52 breaths per minute. Laboratory tests showed anemia (hemoglobin 7.9 mg/dL, hematocrit 22.5%), 42,110 leukocytes/mm^3^ (82% polymorphonuclear), and 899,000 platelets. Inflammatory markers and d-dimer were high (CRP of 27.1 and 5200 ng/mL respectively). Hemoglobin electrophoresis showed hemoglobin (Hb)A1, HbBA2, HbF, and HbS of 46.2%, 2.5%, 19.9%, and 38.4%, respectively. Doppler ultrasound of the left lower limb showed a recent total femoropopliteal venous thrombosis, the head of which was located at the level of the external iliac vein, as well as subcutaneous edema and dermal thickening related to venous obstruction.

Chest X-ray showed retrocardiac pneumonia in the left lung. The patient received oxygen therapy to maintain an oxygen saturation level above 92%, red cell concentrate, cefotaxime for 7 days concomitantly with lincomycin, vitamin C, zinc, and dexamethasone for 5 days, and heparin sodium from the second day of hospitalization.

On the fourth day of hospitalization, the patient remained irritable and his left lower limb painful. C-reactive protein (CRP) rose to 40.9 mg/L. Chest radiograph showed a slight increase in the cardiac area, suggestive of pericardial effusion. Chest tomography could not be done. Nasopharyngeal swabs at admission and on the eighth day of hospitalization, processed at the provincial reference laboratory, did not detect SARS-CoV-2. However, serology tests for SARS-CoV-2 antibodies were positive for IgM and negative for IgG, indicative of COVID-19 seroconversion. White blood cells decreased to 20,730 leukocytes/mL, but CRP increased further to 138 mg/dL. Initial international normalized ratio (INR) of 1.4 and anti-streptokinase antibodies of 91 UI/mL were found. Doppler ultrasonography of the left lower limb showed a partially regressive crural deep vein thrombosis.

On day 10 of hospitalization, the patient was hemodynamically unstable and had classical signs of cardiac tamponade; urgent echocardiography showed a voluminous pericardial effusion, associated with signs of cardiac tamponade. Pericardiocentesis yielded an outpouring of approximately 100 mL serofibrinous exudation fluid. The patient died on day 10 from heart failure. No autopsy was done.

## Discussion

The first severe SARS-CoV-2 infection in children reported from China started with gastrointestinal symptoms, exhibiting no obvious early respiratory manifestations, but progressed rapidly to acute respiratory distress syndrome. Since then, some pediatric case series from China and Europe have been reported with critical illness requiring hospitalization and intensive care support [1].

Since the beginning of the pandemic in the DRC at the end of March 2020, no severe COVID-19 pediatric infections have been described in Lubumbashi, DRC, until the current case.

The clinical course of our patient was atypical, with a painful swelling of the left lower limb as initial symptom, followed by fever, unilateral lower leg swelling, and dyspnea. According to the clinical characteristics of existing pediatric cases, which have been divided into five clinical types, our patient had a severe COVID-19 infection.

The primary results of laboratory investigations suggested an underlying inflammatory phenomenon (CRP of 27.1) and possible predisposition to prothrombotic events (d-dimers = 5200 ng/mL) as described in adult patients. Elevated levels of d-dimer observed at the primary biologic checkup in our patient have been associated with increased severity and mortality in previously reported patients [3]. The prognosis of COVID-19 in patients with comorbidities is known to be poor. For example, although it has been reported that the clinical course seen for sickle-cell disease (SCD) patients with COVID-19 was no different to those without SCD following several case series studies [4–6], although there are some reports that have suggested worse COVID-19 complications in SCD [7–10]. In our patient, hemoglobin electrophoresis showed HBA1, HBA2, HBF, and HBS of 46.2%, 2.5%, 19.9%, and 38.4%, respectively. As a result of the predisposition to a prothrombotic state in patients with SCD and COVID-19, Doppler ultrasonography of the left lower limb showed a femoropopliteal venous thrombosis with complete occlusion of the lumen as well as a subcutaneous edema and dermal thickening related to venous obstruction. SCD patients are prone to a prothrombotic state affecting both the arterial and venous systems [11]. The pronounced inflammation in COVID-19 patients also predisposes them to a hypercoagulable state (COVID-19-associated coagulopathy) with very high mortality rates. Hypercoagulability may affect both the venous (deep vein thrombosis/pulmonary embolism) and arterial systems (ischemic stroke, acute occlusion of the arteries of the limbs, and mesenteric ischemia). Thus, (even heterozygous) SCD patients with COVID-19 may be at high risk of developing thromboembolic events. As previously suggested, our patient was treated with therapeutic doses of enoxaparin (Lovenox) at 1 mg/kg 12 hourly, with a significant regression of the femoropopliteal venous thrombosis within 72 hours of anticoagulant therapy.

Moreover, the patient had lymphopenia and thrombocytopenia, which have been identified as poor prognostic markers in COVID-19 patients [12].

The initial chest radiograph showed a slight increase in the cardiac area suggestive of pericardial effusion and about 10 days later, the patient had classical signs of cardiac tamponade confirmed by an urgent echocardiography showed voluminous pericardial effusion, associated with signs of cardiac tamponade. The American Medical Association, diagnostic Criteria for Acute Pericarditis has been defined as follow: At least 2 of the following clinical criteria are required: Chest pain (typically sharp and pleuritic, improved by sitting up and leaning forward), Pericardial friction rub, Suggestive changes on electrocardiography (widespread ST-segment elevation or PR depression) and new or worsening pericardial effusion Additional Supportive Criteria for Pericarditis has also been suggested: Elevation of markers of inflammation (eg, C-reactive protein), Evidence of pericardial inflammation by an imaging technique such as cardiac magnetic resonance imaging [13].

In our patient, ECG and cardiac RMI wasn’t available and therefore could not be done at the time of his hospitalization in our medical center. However, we have considered clinical biological (CRP) and Echocardiographic features as sufficient to make the diagnosis of constrictive pericarditis in our technologically under-equipped medical centers [14].

The affinity of the SARS-CoV-2 virus for the heart may be explained by the direct binding of its spike protein to human angiotensin-converting enzyme 2, which is present in the human heart, thus allowing cellular infection. It has been shown in COVID-19 that direct pulmonary lesion pathophysiology, viral replication, and dissemination from the seventh day after symptoms onset may lead to a cytokine storm syndrome and direct myopericardial lesion by inflammatory cell infiltration. The fatal outcome and extreme celerity of respiratory and hemodynamic deterioration in this patient reinforce this hypothesis.

Regarding medical care procedures, pediatricians treating patients with COVID-19 are faced with a lack of solid evidence and guidelines regarding the management of COVID-19-related pericardial disease. Nonsteroidal antiinflammatory drugs should not be introduced, considering the risk of respiratory worsening. Treatment is with steroids, and if severe, immunoglobulins have been suggested, following several clinical studies to manage cytokine storm syndrome. Our patient developed severe cytokine storm syndrome and pericarditis despite ongoing high-dose steroid treatment from the second day of hospitalization, suggesting that steroid administration itself could not prevent those critical complications in a patient with hematologic comorbidities. There is growing evidence from ongoing adult studies supporting the use of colchicine as a treatment option in COVID-19-related pericarditis.

## Conclusion

The current case is sufficient to alert pediatricians to the necessity for early identification of children with COVID‐19, especially those with SCD, specifically in countries with high prevalence of sickle-cell disease mutation. As the risks of thromboembolic events and cytokine storm syndrome are high, SCD–COVID19 comorbidity should benefit from early anticoagulation and aggressive antiinflammatory therapies.

## Data Availability

The data that support the findings of this case report are available from the corresponding author upon reasonable request.
